# Supplementary material for: Gender considerations for supportive supervision in humanitarian contexts: A qualitative study
Source: Glob Ment Health (Camb). 2023 Aug 14;10:e50. doi: 10.1017/gmh.2023.33 (PMC10579646; doi:10.1017/gmh.2023.33)
Supplement: O’Sullivan et al. supplementary material [file S205442512300033Xsup001.docx]

**Supplemental Material**

**Semi-Structured Interview Schedule (Combination of interview questions from both first author and second/third authors)**

1. What is your role in MHPSS?

2. What do you understand by the term ‘gender’?

3. What gender elements come to mind when you think about MHPSS supervision?

4. What are some of the gender considerations, if any, to consider when recruiting supervisors?

5. What gender-related factors might hinder someone from becoming a supervisor? Do your supervisors tend to be men, women or mixed? Why do you think that is?

6. In your experience how do family roles help or limit an individual’s involvement in supervision in MHPSS?

7. What are some of the gender considerations, if any, when planning supervisor/supervisee arrangements? How do cultural and gender norms impact supervisory gender pairing and the supervisory relationship?

8. Do gender considerations differ across different supervision formats? If so, how?

9. How do cultural and gender norms impact one’s ability to perform supervisory tasks? To receive training in supervision?

10. Are there any gaps you see within your organisation when it comes to gender considerations?

11. How do you think the IMS could be improved with regards to gender considerations in MHPSS and supervision? *Specific to interviews for participants who engaged in IMS training*
